# Navigating prognostic uncertainty with H.O.P.E.: Caring for elderly patients with chronic kidney disease

**DOI:** 10.1016/j.pec.2026.109567

**Published:** 2026-02-27

**Authors:** Nataly R. Espinoza Suarez, Susan Curtis, Iris Hargraves, Kasey Boehmer, Margaret d’Uscio, Brigid Amberg, Erica J. Sutton, Annika Beck, Annie LeBlanc, Allyson Hart, Jon Tilburt, Bjorg Thorsteinsdottir

**Affiliations:** a VITAM Research Center on Sustainable Health, Quebec Integrated University Health and Social Services Center, Québec, Canada; b Biomedical Ethics Research Program, Mayo Clinic, Rochester, MN, USA; c Knowledge Evaluation Research Unit, Mayo Clinic, Rochester, MN, USA; d Division of Nephrology and Hypertension, Mayo Clinic, Rochester, MN, USA; e Faculty of Medicine, Laval University, Quebec, Canada; f Division of Nephrology, Department of Medicine, Hennepin Healthcare, Minneapolis, MN, USA; g University of Minnesota Medical School, Minneapolis, MN, USA; h Division of General Internal Medicine, Mayo Clinic, Scottsdale, AZ, USA; i Division of Community Internal Medicine, Geriatrics and Palliative Care, Mayo Clinic, Rochester, MN, USA

**Keywords:** Elderly patients, Communication, Prognosis, Chronic kidney disease, Uncertainty

## Abstract

**Objective:**

To explore how older patients with chronic kidney disease (CKD) describe preferences for prognostic conversations.

**Methods:**

We used reflexive thematic analysis to analyze transcripts of semi-structured interviews with CKD patients. Participants were recruited between July 2017 and November 2020.

**Results:**

We identified three major themes: (1) “To Know or Not: Honoring Patient Choices” encapsulates how prognostic conversations should center on the patient’s preferences. (2) “Earlier or Later: Embracing Diverse Perspectives on Timing” underscores how each patient holds a unique perspective on the timing of prognosis discussions, and (3) “Navigating Uncertainty: Humanistic Qualities That Empower Healthcare Professionals to Support their Patients through Prognostic Conversations”, highlights humanistic qualities that enable healthcare professionals to serve as sources of support for patients facing uncertainty during these conversations.

**Conclusion:**

Clinicians should proactively address the uncertainty of CKD by assessing patients’ informational needs, understanding their experiences and values, and gauging their openness to advance care planning. Tailoring communication and fostering a strong clinical rapport can enhance patient engagement and support difficult conversations throughout the disease trajectory.

**Practice implications:**

We propose a mnemonic to help clinicians consider and implement these themes as they empower their patients to make health care decisions in the face of uncertainty. **HOPE**: **H**onoring patients’ needs and wishes, **O**ffering support, **P**romoting connections with careful and kind conversations and **E**xploring feelings and expectations. Clinicians should continue to strive for a strong human connection and provide prognostic information tailored to each patient’s disease trajectory, needs and wishes.

## 1. Introduction

World-wide, about 700 million people are affected by CKD [1] with the majority of affected individuals 65 years of age or older [2]. Chronic kidney disease (CKD) is characterized by a progressive decline in kidney function, often over decades. Disease progression is variable but for some patients, kidney function eventually deteriorates to end-stage kidney disease (ESKD) characterized by heavy symptom burden and life-threatening complications [3,4]. Renal replacement therapies (RRT) for ESKD include kidney transplantation and various forms of dialysis treatments. Alternatively, patients can opt for conservative management without RRT. Dialysis, the most common treatment for ESKD, involves substantial treatment demands and can introduce physical, emotional, and logistical challenges for patients [5,6].

The Renal Physicians Association and the American Society of Nephrology recommended that clinicians fully inform patients with ESKD patients about their prognosis and treatment options [7]. However, clinicians find it challenging to talk about prognosis with their patients. They report struggling to initiate and navigate conversations about diagnosis, prognosis, and progression, and may avoid these conversations altogether [8–11]. Specifically, healthcare professionals report a lack of confidence in communicating uncertainty [12] and feel ill-equipped to break bad news and discuss prognosis or treatment options and fear causing emotional harm through discussion of end-of-life concerns with their patients [13–16].

The inherent uncertainty in prognostic estimates can undermine clinicians’ confidence in their professional competence and hinder transparent communication with patients [12,13]. Furthermore, lack of uniformity in the format, quality, and access to information resources [14] negatively impacts the quality of communication and decision-making support patients receive. Patients with CKD often describe getting insufficient information and support as they learn about their disease and make decisions about complex treatment options [15–17], leaving them uncertain about and at times regretting their choices [8,18].

Prognostic information is important to facilitate shared decision-making and support patient autonomy [19–23]. Most patients with ESKD want prognostic information, but few report that this information is shared with them [11,24,25]. Research shows that a patient’s experience is enhanced when clinicians openly share and explain the uncertainty of prognostic estimation [26,27]. Patients who have discussed prognosis with their clinicians are more prepared for clinical setbacks, less likely to pursue intensive therapies at end of life and more likely to choose hospice [28,29]. Nevertheless, patients – especially the high-risk elderly demographic – report that their goals and values are not consistently prioritized by clinicians [25,30,31].

Healthcare professionals are uniquely positioned to help patients mitigate the ambiguity and weight of prognostic information. Clinicians’ informed recommendations influence patients’ decisions and their confidence in their choices [11,32,33]. Therefore, our objective was to explore the recommendations that individuals with CKD consider helpful for physicians to keep in mind when discussing risks, benefits, and prognoses. Specifically, we sought to identify patients’ communication preferences regarding prognostic discussions, including humanistic characteristics [34], such as respect, compassion, and empathy, perceived to facilitate these challenging conversations. Drawing on these insights, we propose communication strategies to help health care providers better support patients navigating the uncertainties surrounding CKD prognoses.

## 2. Methods

### 2.1. Study design

We conducted semi-structured interviews as part of a larger mixed-methods study, CKD Journeys, between July 2017 and July 2019 [35]. The study was conducted in a multi-disciplinary CKD clinic at a single tertiary referral center in the USA serving local and referral patients. The Mayo Clinic Institutional Review Boards approved this protocol (protocol # 16–005457). We used the Consolidated Criteria for Reporting Qualitative Research (COREQ) framework to guide study planning and reporting [36].

### 2.2. Participant selection and recruitment

We sampled purposively using consecutively screened nephrology clinic patient appointment lists. Participants were 65 years of age or older, living with advanced CKD (Stage 3b or higher), and English speaking. Exclusion criteria included prior dialysis, kidney transplant, or moderate (or greater) cognitive impairment as determined by the Montreal Cognitive Assessment (MOCA) [37]. We called potential participants the day before their clinical appointments to introduce the study. We then met interested patients in the clinic before their appointment to explain aims and get oral consent to undergo the MOCA. Participants with MOCA ≥ 21 gave written consent to participate in the study. We scheduled in-person and telephone interviews within 48 h of the clinic visit. Family members participated in some interviews per the participant’s request and gave oral consent. All participants received a $25.00 gift card for their participation.

### 2.3. Data collection

Three interviewers (AB, SC, NE) conducted in-depth, semi-structured, one-on-one interviews (See Appendix for interview guide). Participants were asked to share experiences and perspectives on receiving prognostic information for CKD and life expectancy, either at the current visit or at any point in their treatment. Irrespective of experience of prognostic disclosure, we asked about the perceived value, benefits and challenges of engaging in such discussions with their clinicians. The interview guide was iteratively modified after early interviews to improve flow and enhance the depth, relevance, and quality of patient responses. After 6 initial interviews, questions were adjusted to be more direct and succinct. Interviews were audio-recorded, transcribed verbatim by a professional transcription service, and compiled with field notes written after each interview.

### 2.4. Analysis

Transcripts were analyzed using inductive coding and a reflexive thematic analysis [38] using NVivo software Version 11 (QSR Intl Inc; Burlington, MA) to assist with data management and analysis. Four members of the research team (AB, SC, NE, BT) developed an inductive codebook based on team discussion around the first four interviews. Later, we conducted line-by-line coding and refined the codebook as necessary throughout the coding process. All transcripts were recorded accordingly. Each transcript was double coded and consensus was achieved. Coding decisions happened in real time, with differences in interpretation resolved through consensus. Thus, codes were refined and adjusted jointly, ensuring conceptual alignment and consistency across the dataset emphasizing reflexive dialog and shared interpretive understanding. After coding, we used memory cards and mapping analysis, engaged in aggregate analysis of coded excerpts, and identified over-arching themes and sub-themes.

### 2.5. Theoretical framework

Given the prominence of uncertainty in participants’ reflections, and clinicians’ perception of prognostic uncertainty as a barrier to disclosure, we drew upon Merle Mishel’s Uncertainty in Illness Theory (UIT) for our analysis. Mishel defines health and illness-related uncertainty as “the inability to determine the meaning of illness-related events and accurately anticipate or predict health outcomes” [39]. UIT posits that factors such as disease complexity, lack of information, and the unpredictability or ambiguity of events can impede patients’ ability to derive meaning from their circumstances, thus heightening their uncertainty [39]. The UIT framework allowed us to deeply understand our participants’ attitudes regarding the uncertainty of their disease progression and how they influence decision making and relationships with clinicians.

### 2.6. Researcher reflexivity

In keeping with our reflexive approach to thematic analysis [38], our subjectivities as researchers productively shaped this project. Of the researchers who directly contributed to coding and analyzing the data, three are trained in the humanities (SC, ES, AB), and three are physicians (NE a family physician, BT a general internist, bioethicist and palliative care physician, and AH a nephrologist). All have experience interviewing or caring for patients with the diagnosis of CKD at an academic tertiary clinic or a safety network hospital reflecting different system realities, patients’ needs and experiences.

## 3. Results

We conducted 28 interviews, lasting between 30 and 60 min. Participants’ age ranged from 66 to 90 years. 68% were men, and 93% were white. Most (69.9%) had adequate health literacy as assessed by a single pre-interview survey item about comfort filling out forms from the health literacy scale [40]. Table 1 shows further details.

Our qualitative analysis identified three themes embedded within and across transcript data. “To Know or Not: Honoring Patient Choices in Prognostic Conversations” encapsulates how prognostic conversations should center on the patient’s preferences. “Earlier or Later: Embracing Diverse Perspectives on Prognostic Timing” underscores how each patient holds a unique perspective on the timing of prognosis discussions, and “Navigating Uncertainty: Humanistic Qualities That Empower Healthcare Professionals in Prognosis Conversations” centers on how clinicians can best support for patients. For quotations supporting each of these themes and subthemes, see Table 2.

### 3.1. To know or not: honoring patient choices in prognostic conversations

This theme examines patients’ preferences regarding prognostic conversations. Participants recommended that patients be allowed to decide whether, and to what extent, they receive prognostic information and insights into the likely progression of their disease “***Asking them how much information they would like or if they would like that information.”*** (Quote 1). Patients’ preferred communication styles differed; for some, a timely, honest, and straightforward delivery was deemed critical ***“don’t beat around the bush”*** (Quote 2 and 3). Other participants suggested a more cautious approach, wanting the clinician to determine what patients need to hear and when based on their perception of their patient’s ability to handle the information “***depending on the frame of mind you were in or the kind of person you were”*** (Quote 4). A subset of patients desired prognostic information as a tool to navigate unpredictability by taking appropriate actions to optimize disease management ***“when you know you try a lot harder”*** (Quote 5). Some thought a prognosis could help patients make decisions, mitigate feelings of uncertainty “***it’ll give me that opportunity to make definite decisions”*** (Quote 6) and plan their new future “***could plan my life around trying to do what I need”*** (Quote 7).

### 3.2. Earlier or later: embracing diverse perspectives on prognostic timing

This theme highlights distinct viewpoints regarding the optimal timing of intricate prognostic discussions. Some participants advocated for early disclosure and ongoing discussions about prognosis throughout a patient’s illness. Early disclosure offers patients more time to both process the diagnosis and treatment options “***I would probably want the information right away so I could think about it*.“** (Quote 8) and take steps that might improve their prognosis and regain control of their health “***so you can do what you can to make things better*.**“ (Quote 9). Others highlighted the crucial role of clinical judgment in guiding the timing of prognostic conversations, preferring a delay in the discussion until clinicians have a clear and accurate prognosis “***Whenever they can give me a reasonable answer of how long I’ve got*.** “ (Quote 10) or disease trajectory “***because I know they assess your general condition and other factors*** “(Quote 11). Some patients insisted clinicians wait until patients explicitly ask for a prognosis to ensure they are ready “… ***don’t give it to ‘em outright [prognosis information]. Wait until they ask, absolutely”*** (Quote 12). Still, some participants – particularly symptom-free participants-struggled to pinpoint the ideal time to discuss prognosis given the unpredictability of the disease “***Um, maybe in the future. Right now, its hard to say. “*** (Quote 13).

### 3.3. Navigating uncertainty: humanistic qualities that empower healthcare professionals to support their patients through prognostic conversations

This theme delineates optimal approaches to communication, prioritizing the patient’s overall well-being. Participants identified specific qualities that enable healthcare professionals to serve as valuable sources of support in prognosis conversations. Patients underscored the importance of developing strong rapport before difficult conversations about CKD treatments and prognoses “***I think being able to have a good rapport with a physician is one of the first pillars of building that communications bridge*** “ (Quote 14 and 15). Certain qualities in clinicians were perceived to demonstrate support, help foster trust, and instill feelings of security while navigating CKD diagnosis and prognosis. For example, participants wanted clinicians to convey prognosis information truthfully and compassionately “***a doctor that is for the patient regardless and for the doctor to be honest with me. “*** (Quotes 16 and 17). They felt clinicians should exhibit sincere care and dedicate time to listening attentively “***that you’re not being hurried in and hurried out, that is a nice trait***.” (Quote 17). Participants wanted to feel cared for by someone genuinely interested in their well-being “***that you’ve got a concern for the patient’s mental wellbeing.”*** (Quote 18). Participants also felt clinicians should exhibit humility and directness by using lay language to enhance patient understanding “***Just to talk like a normal person. You know, don’t use them big words and stuff*** “ (Quote 19). Essentially, participants saw clinicians as potential allies in their efforts to remain as healthy as possible, seeking not only clinical information, but also support, encouragement, and hope as they assimilate prognostic information into their new health narratives “. ***I mean keep a positive attitude. Give ‘em a positive attitude.“*** (Quotes 16 and 20).

## 4. Discussion and conclusion

### 4.1. Discussion

#### 4.1.1. Summary of findings

Prognostic communication in chronic illness represents a delicate balance between uncertainty and hope, where the clinician’s ability to acknowledge ambiguity while sustaining meaning and emotional connection becomes central to patient-centered care.

The themes **“To know or not”** and **“Earlier or Later”** illustrate the diverse ways in which patients respond to prognostic disclosure. These variations highlight the need for **individually tailored prognostic conversations**, which can help patients navigate the uncertainty inherent to CKD, even when new ambiguities arise from prognostic estimates. This interpretation aligns with the **Uncertainty in Illness Theory (UIT)**, which explains how unpredictable disease trajectories and limited information amplify uncertainty, influencing whether patients seek or avoid prognosis-related knowledge. Within this context, **clinicians play a crucial role in fostering hope** and supporting patients’ understanding of their condition. As emphasized in *“Navigating Uncertainty: Humanistic Qualities That Empower Healthcare Professionals in Prognosis Conversations,”* the effectiveness of such dialog depends greatly on the **clinician’s humanistic approach and communication style**.

Ultimately, addressing uncertainty with compassion and a focus on hope enables patients to make informed decisions about their health without feeling overwhelmed by fear [41–43].

To deepen the interpretation of our findings, we drew upon **Mishel’s Theory of Uncertainty in Illness** as a conceptual lens to examine how patients navigate ambiguity, information needs, and emotional responses during prognostic conversations. Guided by this theoretical framework and our inductive thematic analysis, we transformed participants’ narratives into actionable insights for clinical communication. Through this integration of empirical and conceptual work, we developed the **HOPE mnemonic**—a practical tool designed to propose clinicians an approach prognostic uncertainty with empathy, openness, and patient-centeredness. HOPE encapsulates four key principles: **Honoring** patients’ needs and wishes, **Offering** support, **Promoting** connection through careful and compassionate dialog, and **Exploring** feelings and expectations. Although not a validated framework, HOPE represents a thoughtful synthesis of our qualitative findings and theoretical reflections, offering a foundation to enhance communication and quality of care in the context of chronic illness.

#### 4.1.2. Honoring patients’ needs and wishes

While nephrologists acknowledge the importance of patient values and preferences [44,45] previous studies have suggested that they may have limited working knowledge of what patients deem significant in their lives [46]. Treatment recommendations are often primarily based on biomedical factors with a bias toward preserving life [44,45,47, 48–50]. Some patients prefer detailed discussions about prognosis, while others focus on day-to-day well-being [21]. Supporting these diverse preferences with conversations that center on what matters most to the patient allows them to make informed choices about their care [21].

Our findings align well with that of other experts in shared decision making around ESKD treatment. The **NephroTalk training** emphasizes shifting from a disease-centered to a patient-centered focus [51]. Similarly, the **HIGHWay initiative** (“Honor Individual Goals and Hopes Way”) operationalized this principle by integrating advance care planning into dialysis care through structured, values-based discussions [52].

These programs echo the central tenet of our findings: honoring patients’ wishes requires both skill and structure. By embedding such frameworks into clinical practice, clinicians can move beyond hesitancy toward proactive, values-driven dialog.

Furthermore, by prioritizing shared decision-making and respecting patients’ autonomy, clinicians create a care environment that empowers them to navigate their illness in a way that aligns with their values and needs [53].

#### 4.1.3. Offering support

When patients encounter uncertainty with their prognosis or treatment plans, clinicians are a source of support. Creating a safe and compassionate space for patients to articulate health-related concerns and personal fears can alleviate distress associated with CKD and strengthen the therapeutic alliance with their clinician [54]. This also enables clinicians to design treatment plans that increase the likelihood treatment experiences will match patient’s preferences. treatment experiences that patients most wish to avoid. Utilizing a structured communication guide or checklist can help clinicians explore patients’ priorities, clarify prognosis, enhance clarity and quality, and ensure that emotional and existential concerns are addressed [46,55]. The Serious Illness Conversation Guide pilot study (2023) demonstrated that structured communication frameworks help clinicians navigate complex conversations effectively. The use of standardized prompts improved clinicians’ confidence and ensured that critical topics—such as values, fears, and trade-offs—were discussed consistently.

Patients reported feeling “heard” and “supported,” underscoring the relational dimension of offering support. Similarly, the HIGHWay initiative [52] emphasized the importance of continuity and follow-up, where repeated, iterative conversations allowed patients to reflect on evolving goals, reaffirming that support is a process rather than a single event.

Another supportive approach is to encourage patients to involve their broader social support networks. This allows clinicians to identify sources of aid in coping and demonstrates respect for the patient’s values, particularly crucial for Black patients [56,57]. As preferences for family involvement vary widely, clinicians should seek explicit consent and balance autonomy with relational support [58]. Older patients often care deeply about the effects of their illness on their loved ones [47,59]. It is important for care teams to facilitate recognition of the patient’s expected clinical trajectory and address concerns about burdening family members and facilitating their engagement can alleviate patient anxiety and improve family coping and bereavement outcomes [31]. Finally, patients and their families need to understand the uncertainty of prognostic assessments. As previously reported, participants in our study were accepting of prognostic uncertainty and felt it could be leveraged for hope and motivation to achieve their health goals [21].

#### 4.1.4. Promoting connections with patients with careful and kind conversations

Our participants also stressed the importance of clinicians customizing information to their preferences, a sentiment echoed in studies finding that *tone* and *manner* of conversations were as critical as the factual content, and that delivery profoundly shapes patients’ understanding and comfort [11,51,52,58].

Our participants expressed a preference for strong clinician-patient relationships before engaging in prognostic discussions, consistent with the findings of others who reported a preference to have such conversations with clinicians who knew them well [58]. Similarly, relationships and trust were essential for meaningful advance care planning, and alignment of expectations [52] This preference is consistent with prior studies that have highlighted CKD patients’ desires to discuss goals of care and advanced directives with trusted individuals who are familiar with their medical history, such as their nephrologists, primary care physicians, or social workers [60]. Within these relationships, prognostic conversations offer opportunities not only to discuss timelines, but also to address expected symptom burden [61], functional changes {Michel, 2005 #283}, risks of hospitalization, and potential care needs over time [62]. Importantly, participants’ interests in prognostic information extended beyond life expectancy and often centered on what the future might entail, including the anticipated disease trajectory, the timing of key transitions such as dialysis initiation, and the potential impact of treatments on daily life and functioning {Thorsteinsdottir, 2022 #284}[63]. Taken together, these conversations provide an important opportunity for clinicians to deepen their understanding of patients by exploring their values, concerns, fears, and priorities for the future [60,64–66].

#### 4.1.5. Exploring feelings and expectations

Coping with uncertainty is difficult, but prognosis discussions are easier when clinicians understand patients’ perspectives. In our study, participants expected clinicians to demonstrate curiosity and actively seek information about their feelings regarding the future. Meuleman et al. similarly emphasize the importance of assessing patients’ concerns about their illness, their beliefs about controllability, and their understanding of their condition [27]. Clinicians cannot assume that patients are coping well or fully understand their disease or treatment simply because they do not voice their struggles. They should explore illness views, information preferences, emotions, and care expectations early and regularly. This finding is significant considering O’Hare et al.’s finding that when providers demonstrate inadequate insight into or concern for patients’ experiences of illness, it can lead to feelings of mistrust, abandonment, isolation, alienation, and the deterioration of the clinician-patient relationship [67].

#### 4.1.6. Limitations and strengths

Since we sampled from a tertiary referral center in Minnesota, our findings may not be transferable to other healthcare settings, despite our robust sample size. Our rigorous approach to qualitative analysis and interpretation, including double coding each transcript and triangulation with a multidisciplinary study team, enhances the trustworthiness of our interpretation.

Findings were shared with patients from a diabetic kidney disease patient advisory group and with clinician focus groups. Both groups agreed with our interpretations, results, and conclusions, supporting the credibility of our findings. These discussions however, did not include a presentation of the HOPE mnemonic itself. The HOPE mnemonic, emerged as a conceptual and practice-oriented tool derived from the data, informed by our data and theoretical orientation. HOPE has not yet been validated but rather emerged as a conceptual and practice-oriented tool derived from the data. In line with the ethos of qualitative research, it is both appropriate and valuable to develop concepts inductively, drawing directly from participants’ narratives and lived experiences, as it allows ideas to emerge organically from participants’ stories and lived realities.

Future research should explore and validate the applicability and transferability of the HOPE mnemonic across diverse clinical and cultural contexts. Its relevance and robustness could be enhanced through future studies that build on this work and assess the mnemonic’s applicability and transferability across different clinical and cultural settings.

## 5. Conclusion

Uncertainty is among the most common challenges patients face upon receiving a potentially life-threatening diagnosis [68]. CKD trajectory in particular is unpredictable and uncertain. Therefore, clinicians should anticipate and react to changes in the clinical trajectory and proactively inquire whether patients want, have or do not desire information. Additionally, clinicians should determine each patient’s understanding of and personal experiences with kidney disease, explore their values around quality of life, and gauge their receptiveness to advance care planning. Knowing patients’ expectations allows clinicians to tailor information sharing, engage patients as partners, and cultivate a clinical rapport that can support difficult conversations throughout the course of disease.

### Practice implications

Our findings describe how clinicians could engage in supportive prognostic conversations with their patients. Resources exist to help clinicians navigate and communicate uncertainty while demonstrating commitment to patient care [69]. Patients should receive prognostic information sensitively, in alignment with their needs and preferences, to foster informed decision-making and quality of life. To help clinicians support patients facing the uncertainty of their CKD disease progression, we propose clinicians consider the four components captured in the mnemonic H.O.P.E.

Improved institutional and educational efforts are needed to support patient-centered best practices for prognostic conversations within the CKD population. Consistent with the goals of the NephroTalk program, our data confirm that patients want clinicians to effectively recognize and respond to patients’ emotional cues (e.g., acknowledging distress, naming emotions, and expressing empathy), thus fostering a sense of being supported among patients. Providing education and decisional support in a timely manner facilitates effective decision-making, potentially averting treatment delays linked to heightened patient morbidity, mortality, and healthcare expenses [70]. Given the complexity of engaging in such conversations [71], we suggest the H.O. P.E. mnemonic to foster supportive prognostic conversations and strengthen patient-clinician connections. Future research should focus on implementing these recommendations and identifying system-level changes to enhance patient-centered discussions. Patient involvement remains crucial in developing tailored frameworks. Insights from our study inform *CKD Journeys*, a decision aid currently in pilot testing. This manuscript aims to guide clinicians in navigating these essential yet complex conversations.

Table 3 translates the four components of the H.O.P.E. mnemonic into concrete, actionable recommendations for clinicians and educators. By illustrating how to recognize readiness, respond empathetically, foster shared understanding, and tailor communication to patients’ literacy, emotions, and cultural contexts, the table functions as a proposal to both a practical guide for clinical encounters and a pedagogical tool. In practice, it provides a structured yet adaptable framework to support meaningful, patient-centered prognostic discussions. In education, it can inform communication training and reflective exercises aimed at cultivating key relational skills—listening, empathy, and emotional attunement—often overlooked in nephrology curricula. Thus, Table 3 represents a tangible contribution of this study to advancing compassionate, patient-centered communication in chronic illness care.

## Supplementary Material

Appendix

## Figures and Tables

**Table 1:** Participant demographic characteristics 5 of the participants did not return the survey.

	Patient n = 28
Sex (F)	9
Age Mean (SD)	77.7 (6.8)
Age Range	66–90
White race (%)	25 (89.3)
**Health literacy adequate**	18 (64.3)
**Marital Status**	
Married	13
Divorced	2
Widowed	8
Never married	2
**Residence**	
Minnesota	18
Non-Minnesota	10
**CKS stage**	
Stage 3	6
Stage 4	15
Stage 5	7

**Table 2:** Explanatory quotes for each theme.

Theme	Quotes
**To Know or Not: Honoring Patient Choices in Prognostic Conversations**	1. ***Asking them how much information they would like or if they would like that information. We can do this ‘cuz they might not even think about statistics and so forth. You could say ‘We can give you this information’, but then ultimately, it would be their choice whether they wanted to hear it or not*.** (Patient 11, 79 years)
	2. ***When a patient, if a patient’s got bad labs and their GFR is falling and they’re in line for a kidney dialysis, then tell ‘em. Don’t lie to ‘em. Don’t hold back. Just give it to ‘em straight and then give ‘em ideas of how we’re gonna fix it, okay. Don’t say “well, I’m not sure now”. No. That’s not acceptable*.** (Patient 20, 69 years)
	3. ***Give me bad news, if he’s got bad news, give it to me. Don’t beat around the bush. Tell me. Yeah. Tell me what’s good, what’s bad for me. That’s all I need*.** (Patient 05, 80 years)
	4. Interviewer: …what advice do you have for them about how to talk to patients and how to present this? Do you have some advice for us? ***Patient: Well, just be honest. Answer questions honestly. Whatever people want, the information they want, give it to them. Probably, maybe to some extent, not any more than they need without asking. You could come here, depending on the frame of mind you were in or the kind of person you were, and leave awful depressed. Like, ‘Oh, this is gonna be what the future is’ and stuff. There’s a line in there when you tell them what they need to know, but only if they really need to know it*.** (Patient 11, 79 years)
	5. 5. Interviewer: Do you have any concerns about talking about these risks or your prognosis with your doctor? ***Patient: I just think it’s good to know because I think when you know you just try that much harder to do the things that you’re supposed to do to just make sure you’re trying to do things right. (Patient 02, 73 years)***
	6. 6. Interviewer: If we could predict your prognosis based on your circumstances, and disease stage, and lab work, is that something you would want to know? **Patient: *Oh yeah, I’m sure if it can be predicted, I mean, it’ll give me that opportunity to make definite decisions*.** Interviewer: Do you think it’s appropriate—or when do you think—if a doctor or a nurse can do that kind of calculation, when do you think they should offer that to people? **Patient: *I think the sooner the better because I think it’d give*—*it’d be the sooner the better really. Because then their final decision can be made. (Patient 04, 81 years)***
	7. 7. Interviewer: what would be helpful for you to know in order to plan and decide how to deal with your kidney disease? **Patient: *well, yeah. If I had this information that kind of, I guess you’d say being able to look into the future, a precursor type thing, I could plan my life around trying to do what I need with my kidneys, and still try to be as functional farming as I could*.** (Patient 23, 76 years)
**Earlier or Later: Embracing Diverse Perspectives on Prognostic Timing**	8. Interviewer: Some people say they’d like to know right away. Other people aren’t interested in knowing it at all. They would prefer to not have that. **Patient: *oh, I would probably want the information right away so I could think about it*.** Interviewer: What kind of things would you consider when you were thinking about it? **Patient: *Just whether I would want dialysis or not. I’ve not heard very good reports from some people who don’t like it, that its uncomfortable or whatever*.** (Patient 06, 84 years)
	9. Interviewer: Do you have any concerns about receiving prognosis information? **Patient: *no. I think its just good to know these things because, the reason I say that is because I went to my regular family doctor and my kidneys were giving me problems and she never told me anything about it, which I think is very, very wrong. I think that’s why even with dialysis and exactly how things are going, I think you should just always know so you can do what you can to make things better*.** (Patient 02, 73 years)
	10. ***Whenever they can give me a reasonable answer of how long I’ve got before I’d have to go into that servicing [dialysis]*.** (Patient 06, 84 years)
	11. Interviewer: When do you think would be a good time to get that kind or prediction [from clinicians]? **Patient: *I think the patient’s general health plus the changing components of the blood would determine when would be a good time to start. The rate of decline in everybody is going to be a little bit different, so that would be analyzed by the nephrology team. Let them be the main guide to when you should start, because I know they assess your general condition and other factors*.** (Patient 10, 77 years)
	12. **Patient:** [When talking about provider initiated prognosis conversations] ***No, no, you’d freak ‘em out*.** Interviewer: So you just don’t even broach it? **Patient: *You absolutely*—*no*.** Interviewer: Okay. ***Patient: You’d absolutely freak ‘em out. You wait until they’re talking—you know and they’re meeting with the doctor and if he suggests in his diagnosis or whatever, saying, “You know, we don’t have a lot of time,” then they may ask you, “Well, how much time do I have?” or “What will happen?” or “What do I need to do?” That’s when they’re interested. That’s when they’ll listen to you. That’s when they’ll remember it, but don’t give it to ‘em outright. Wait until they ask, absolutely*.** (Patient 20, 69 years)
	13. Interviewer: When might be a good time to get a prognosis prediction? **Patient: *Probably if I start getting sicker. If I could really start feeling like I was getting sick or my kidneys were startin’ to fail, then I think it’d be the time to see how bad they are*.** Interviewer: And say you went in for your next appointment, a routine visit, would this be something you’d want to hear? ***Patient: Um, maybe in the future. Right now, its hard to say. When I’m feeling as well as I am now its hard to say, you know*.** (Patient 18, 67 years)
**Navigating Uncertainty: Humanistic Qualities That Empower Healthcare Professionals in Prognosis Conversations**	14. ***I think part of it [comfort discussing prognosis] is the fact that we have a good relationship, and I can say anything to him. I think being able to have a good rapport with a physician is one of the first pillars of building that communications bridge*.** (Patient 30, 70 years)
	15. Interviewer: From your experience, do you have recommendations for doctors and nurses to keep in mind when they’re trying to help people understand kidney disease and what these different options are, and the life expectancy? **Patient: *You have to ease into the relationship, and know the patient, how much you can tell them about the severity or the probable outcome*.** Interviewer: Okay, ease into things. Information you think is very helpful **Patient: *Oh, yeah*.** (Patient 10, 77 years)
	16. Interviewer: We were talking about risks. How should doctors talk about risk, benefit, prognosis with patients, in your experience? **Patient: *I wanna see a doctor that is for the patient regardless and for the doctor to be honest with me*.** (Patient 05, 80 years)
	17. Interviewer: Good. Okay, that’s great. Actually you’re going in a direction of one of the sorts of things that we’re interested in, which is, what are traits of a nurse or doctor that are important to you when it comes to communication? **Patient: *Well, I think caring. When you get the feeling that they’re caring, that they are concerned. I think that’s one trait they have. When you get the feeling that you’re not being hurried in and hurried out, that is a nice trait. Personally the fact that they care*.** (Patient 20, 69 years)
	18. **Patient: *I would think that you’ve got a concern for the patient’s mental wellbeing. To reassure them and not frighten the patient and not scare the patient and say “hey, you’re gonna die tomorrow if you don’t have this [treatment]*.** (Patient 10, 77 years)
	19. **Patient: *Just to talk like a normal person. You know, don’t use them big words and stuff, ‘cause I don’t understand all that stuff. Yeah. Just speak to me like a normal, everyday person. That’s about it*.** (Patient 12, 78 yers)
	20. ***I mean keep a positive attitude. Give ‘em a positive attitude. Give ‘em something’ to grab onto. Give ‘em hope to grab onto*.** (Patient 20, 69 years)

**Table 3:** **H**onoring patient’s needs and wishes**, O**ffering supportive care**, P**romoting connections with careful and kind conversations, and, **E**xploring feelings and expectations **(H.O.P.E.): Recommendations for clinical practice**.

H.O.P.E recommandations	Examples
**Honoring patients’ needs and wishes**	***Respect patients’ readiness, preferred timing and willingness to talk about prognosis*.** Assess patient readiness to talk about prognosis during clinical encounters, using explicit invitations that signal openness to discussing the future.
	*Some people prefer to talk about what may lie ahead, while others would rather focus on the present. How do you feel about discussing what the future might look like right now?* Respect patients’ silence and space Assess patients preferred communication style:*Some people like the whole picture of what to expect down the line laid out for them. Other people feel that is overwhelming and want just the information they need in the next few weeks or months. How do you like to get information about your health?****Provide full disclosure* about [when desired] …** treatment options non-pharmacological treatment options support resources education resources advance directives and end of life discussions
**Offering support**	Acknowledge your patients’ emotions *It sounds like you are feeling*_____, *is that right? This diagnosis would be challenging for anyone, but we can work together to find a treatment plan that works for you*.
**Promoting connections with careful and kind conversations**	***Set the stage by putting patients first*:** Take time Listen and learn Be emphatic Be careful and kind Do not hurry conversations Provide space for questions Answer all patient questions
	***Getting to know your patient*:** Ask about priorities: *“What are the biggest priorities in your life right now?”* Ask about disease awareness: *“What do you want to know about your chronic kidney disease?”* Ask about their feelings: *“How do you feel about prognostic information?”* Ask about their concerns: *“What is concerning you about your chronic kidney disease?”* Ask about social support: *“Do you have people you can ask for support? Do you have the help you need to cope with your kidney disease?”* Ask about advance care planning: *“How might your treatment plan change if your kidney disease progresses?”* Ask about what and how much they want to know about prognostic information: *Are you ready to talk about prognosis?*
**Exploring feelings and expectations**	***No patient is the same*:** Explore your patient’s desire to know about their prognosis. *What information do you need to handle your chronic kidney disease?* *Would you like to talk about your prognosis, or how I think your chronic kidney disease will affect your body over time?* Tailor information to patients’ literacy about their disease and prognosis. Use kind, lay language, and avoid medical jargon. Ask about how patients feel about their diagnosis, or if they have any feelings about the disease progression. *How are you feeling about your diagnosis?* *I want to help you on this journey; are there any fears or concerns you have about this disease that we can talk about?* Ask about what they expect from their treatment. *Please tell me what you know, if anything, about how kidney disease is treated?* Ask explicitly about patient hopes and goals for the future. *When you think about the future, what are you hoping for?* *Are there goals, priorities, or outcomes that matter most to you as we plan your care? Explore hopes openly, recognizing that they may range from highly optimistic expectations to concrete, personally meaningful goals that can inform treatment planning*. Ask about what they expect from the clinical team and how they can help or guide them. *How can I, and members of my team, support you as you deal with this disease?* Adjust and react to patients’ emotional responses.

## Appendix A. Supporting information

Supplementary data associated with this article can be found in the online version at doi:10.1016/j.pec.2026.109567.
