# Supplementary material for: Navigating prognostic uncertainty with H.O.P.E.: Caring for elderly patients with chronic kidney disease
Source: Patient Educ Couns. Author manuscript; Available in PMC 2026 Apr 3. (PMC13046034; doi:10.1016/j.pec.2026.109567)
Supplement: Appendix [file NIHMS2160660-supplement-Appendix.docx]

**Interview Guide**

**ESRD Decision Conversations**

For use with patients and/or surrogate decision makers who have ESRD and may consider dialysis or have made a treatment option decision

Time: immediately following clinical visit with nephrology professional (Mayo 19/Access Clinic) or by phone within 1 week of this conversation

Interview to last 30- 45 minutes

**Introductory Script:** *Thank you for participating in our study. We plan to use what we learn from you and other patients and families like yours to develop decision aids that will make it easier for patients to share their hopes and goals for treatment with their doctors and for doctors to communicate more clearly about the complicated options that are available. This way each patient can make the best decision for them.*

*This should take 30-45 minutes. I’m going to ask a series of questions, if any of them make you uncomfortable please let me know and we can stop. Also let me know if I am not being clear.*

*So I can recall your thoughts more accurately, may I have your permission to record our conversation? All of the information you provide will be kept strictly confidential as we described when you signed the consent. And finally don’t worry about being critical or blunt, we want your honest opinions. Your answers are going to be used by researchers to help develop a decision aid for use with future patients, but they will not be shared with your doctor or have any impact on your care.*

*Do you have any questions before we begin?*

**If permission is granted, interviewer turns on digital recorder.**

*DIALYSIS RISK and OPTIONS*

*Based on your current understanding of your disease, how comfortable are you in choosing a treatment option if your disease progresses?*

*What are the most important considerations or pieces of information to help you make a decision on an option for treatment?*

*There is a calculator that helps calculate the likelihood of needing dialysis at 2 and 5 years.*

*IF patient has had risk predication; ask do you remember your risk? How does it feel to you? Do you think it is accurate?*

*How would you feel about being offered that information?*

*When would it be appropriate to offer this information?*

*How might you use this information?*

*What is the potential value of this information?(To you, to your family)*

*Do you have any concerns about receiving such information?*

*Do you know about the possible side effects of dialysis?*

*Tell me about any side effects of treatment you think would be important to talk about when making decisions about treatment with your doctor.*

*What are things you worry about with different treatment options? (Risks?)*

*What are some things you have wondered about but haven’t asked?*

*Each treatment option comes with some tradeoffs, side effects or other factors that can be a burden to you. Are there some tradeoffs or burdens you are not willing to accept in spite of the benefits of the treatment option?*

*(“in previous studies patient have expressed the wish to hear what to expect in the future given their stage of kidney disease but doctors have expressed concerns or hesitancy to share this information”)*

*PROGNOSIS*

*What is your understanding of your risk of dying with your kidney disease? ( add: how did you come to this understanding?)*

*If we could predict your risk of dying based on your circumstances, disease stage and lab work,*

*How would you feel about being offered that information?*

*When would it be appropriate to offer this information?*

*What amount of time from now would like to this to be measured in? (Are you interested in knowing the predicted prognosis in the short term (6months or less) or longer term (2years, 5 years) or both?)*

*What would be the potential value of such information? (To you, to your family)*

**Final questions**

*Doctors often hesitate to share risk predications or risk of dying predictions with patients and families because there is no way to be 100% accurate. How do you feel about getting a predication that may not be correct?*

*What are important things to think about and talk about in a conversation with a kidney doctor?*

*Do you have any concerns about taking about needing dialysis /risk of dying with your doctor?*

*Have you discussed the risk of death (prognosis) with and without treatment (if not mentioned by patient already)?*

*How did that conversation help you understand your risk/prognosis?*

*From your experience, do you have recommendations you think it would be helpful for doctors to keep in mind when they help people to understand risks and benefits/prognosis?*

***END***
